# Role of T198 Modification in the Regulation of p27^Kip1^ Protein Stability and Function

**DOI:** 10.1371/journal.pone.0017673

**Published:** 2011-03-14

**Authors:** Monica Schiappacassi, Sara Lovisa, Francesca Lovat, Linda Fabris, Alfonso Colombatti, Barbara Belletti, Gustavo Baldassarre

**Affiliations:** Division of Experimental Oncology 2, Centro di Riferimento Oncologico, National Cancer Institute, Aviano, Italy; University of Medicine and Dentistry of New Jersey, United States of America

## Abstract

The tumor suppressor gene p27^Kip1^ plays a fundamental role in human cancer progression. Its expression and/or functions are altered in almost all the different tumor histotype analyzed so far. Recently, it has been demonstrated that the tumor suppression function of p27 resides not only in the ability to inhibit Cyclins/CDKs complexes through its N-terminal domain but also in the capacity to modulate cell motility through its C-terminal portion. Particular interest has been raised by the last amino-acid, (Threonine 198) in the regulation of both protein stability and cell motility.

Here, we describe that the presence of Threonine in position 198 is of primary importance for the regulation of the protein stability and for the control of cell motility. However, while the control of cell motility is dependent on the phosphorylation of T198, the stability of the protein is specifically controlled by the steric hindrance of the last amino acid. The effects of T198 modification on protein stability are not linked to the capacity of p27 to bind Cyclins/CDKs complexes and/or the F-box protein Skp2. Conversely, our results support the hypothesis that conformational changes in the disordered structure of the C-terminal portion of p27 are important in its ability to be degraded via a proteasome-dependent mechanism. On the other hand T198 phosphorylation favors p27/stathmin interaction eventually contributing to the regulation of cell motility, supporting the hypothesis that the presence of T198 is fundamental for the regulation of p27 functions.

## Introduction

The Cyclin Dependent Kinase Inhibitor (CDKI) gene p27^Kip1^ (hereafter p27) is frequently altered in human cancer and its low expression is often associated with a worse prognosis for the patients [1]. Recent evidences demonstrated that p27 could also be functionally altered in human tumors [2]. Although the regulation of p27 could in some settings occurs at transcriptional level [2]–[3], it is widely accepted that in human tumors its expression levels are mainly regulated by post-transcriptional modifications [2]. In particular it has been shown that p27 expression is regulated by a proteasome-dependent degradation [4] and that this mechanism plays a pivotal role in the regulation of p27 expression in several type of human tumors, as first demonstrated in breast and colon cancer [5]–[6]. The proteasome dependent regulation of p27 requires its phosphorylation on the Threonine 187 (T187) by CDK2-containing complexes. This phosphorylation is necessary for the binding of p27 to the F-Box protein Skp2 [7]–[8] and for its consequent ubiquitination and proteasome-dependent degradation [reviewed in 9]. More recently it has been shown that the Skp2-mediated degradation of p27 occurs predominantly during the S phase [10] in accord with the fact that it requires the activity of the CDK-containing complexes [11] and a direct phosphorylation on multiple tyrosine residues [12]–[13]. The overall scenario depicted with new available data is that, starting from a state of quiescence in which the cell has high levels of p27 protein, after growth factor stimulation, p27 is first phosphorylated on three different tyrosine residues (Y74, Y88 and/or Y89) by different intracellular tyrosine-kinases (i.e. c-Src, Bcr-Abl or Lyn, depending on the cellular context). These phosphorylations allow the CDK2 containing complexes to phosphorylate the T187 in p27 eventually resulting in Skp2 binding and ubiquitin-dependent degradation [12]–[13].

Although this pathway has been widely demonstrated and seems to play a pivotal role in the control of p27 protein stability in both normal and cancer cell, at least two Skp2-independent pathways have been proved to induce p27 degradation during G1: the KPC1-KPC2 ubiquitin-ligases [14] and the activation of the Wnt-Cul4A pathway [15]. Both of these pathways could be involved in the regulation of p27 in human cancer favoring cell proliferation and tumor progression. However, the modification necessary for p27 to become a target of either Cul4A or KPC1/2 ubiquitin ligases are still not identified. Interestingly, the phosphorylation of p27 on its last residue threonine 198 (T198) by different kinases [16]–[17] has been proposed to control not only its stability [18] but also to modulate its activity in the regulation of cell motility [17].

Most part of the recent literature suggests that in the N-terminal portion of the protein resides the ability of p27 to bind and inhibit the activity of several cyclin/CDKs complexes while its C-terminal domain seems to control protein stability, nuclear localization and binding to non-CDKs complexes [2].

We previously demonstrated that the last 28 amino-acids in p27 are not necessary for the control of cell proliferation but they are important in the regulation of cell motility [19]–[20]. Since the last 28 amino-acids of p27 comprise both the T187 and the T198 residues, we asked whether a point mutation in these two key threonines could recapitulate the effects of the deletion of the last 28 amino-acids. By generating these two mutants we observed that the T198A mutation determines a drastic reduction of p27 protein expression as previously reported by others [18]. However, our data demonstrate that this effect is not due to the absence of phosphorylation but rather to the steric hindrance of the last amino-acid, indicating that changes in the conformation of the C-terminal tail of p27 render the protein more prone to its proteasomal degradation. Conversely, our experiments of cell motility indicate that T198 phosphorylation is specifically necessary for p27 to function as a modulator of cell motility, favoring its binding to the microtubules destabilizing protein stathmin.

## Materials and Methods

### Cell culture, Transfection and Treatments

HT-1080 fibrosarcoma cells (ATCC CCL-121), HEK 293 (ATCC CRL- 1573) and 3T3 p27 null (p27^KO^) fibroblasts were grown in DMEM supplemented with 10% heat-inactivated FBS (Sigma). U87MG malignant glioma cells (ATCC HTB-14) and MDAH 2774 ovarian adenocarcinoma cells (ATCC CRL-10303) were maintained in RPMI-1640 medium supplemented with 10% heat inactivated FBS. SCC9 squamous cell carcinoma cells (ATCC CRL-1629) were grown in a 1∶1 mixture of DMEM and HAM'S F12 supplemented as described by the provider. The cells were transfected with the indicated expression vectors using FuGENE HD Transfection Reagent (Roche). When indicated, cells were treated with 10 µg/ml cycloheximide (Sigma) for 3, 6 and 9 hours and with 50 µM MG132 (Calbiochem) for 6 and 9 hours.

### Construction of Expression Vectors

The mutants of p27 were generated by PCR on p27^wt^ vector using oligonucleotides carrying the indicated mutations. Amplified fragments were cloned into pGEM-T Easy Vector (Promega) and checked by sequencing. The expression vectors utilized were pEGFP-C (Clontech), pFLAG-CMV 6 (Sigma), pDNR-CMV (Clontech), pQE-30 (Qiagen) and pTNT (Promega). In the same way stathmin coding sequence was cloned in the DsRed-Monomer-C expression vector (Clontech).

### Recombinant Adenoviruses

Recombinant adenoviruses for p27 overexpression were produced with two systems: the Adeno-X Tet ON Expression System 2 (Clontech) and the Adeno-X ViraTrak ZsGreen1 Expression System 2 (Clontech), according provider's instructions. To obtain regulated p27 expression, in the first method target cells were co-transduced with two viruses: the AdTet-ON (Clontech), that provides the constitutive expression of the reverse tetracycline-controlled transactivator (rtTA), and the AdTRE that encodes for the p27^WT^ or mutant proteins. Recombinant protein production was induced using Doxycycline (Sigma) at 1 µg/ml added to cell culture medium. In the other system the gene of interest and the gene for the green fluorescence protein are under the control of distinct CMV promoter, allowing the simultaneous but not fused expression of the two proteins. The control (AdNeg, Clontech) and Skp2 siRNA adenoviruses were generated using the BD Knockout Adenoviral RNAi System 2 (Clontech) following the manufacturer's procedures and inserting the appropriate target sequence: human Skp2: 5′-GCCTAAGCTAAATCGAGAGAA-3′ (NM_032637, pos nt 428–449). Adenovirus production, titration and characterization were performed as previously described [20].

### Expression and Purification of Recombinant Proteins

Production of bacterial recombinant proteins was performed essentially as previously described [19]. Briefly, p27^WT^ and p27 mutants cDNAs were cloned in the pQE-30 vector (Qiagen). *Escherichia coli* (M15) cells were transformed with the expression plasmid to produce recombinant proteins containing His-tag at the N-terminus. Proteins were subsequently purified with Ni-nitrilotriaceticacid (NiNTA) resin (Qiagen).

### Preparation of Cell lysates, Immunoblotting, and Immunoprecipitation

Cell lysates were prepared using cold NP-40 lysis buffer (0.5% NP-40, 50 mM HEPES [pH 7], 250 mM NaCl, 5 mM EDTA, 0.5 mM EGTA [pH 8]) plus a protease inhibitor cocktail (Complete, Roche), 1 mM sodium orthovanadate, and 1 mM dithiothreitol as previously reported [19], [21]. Differential extraction of nuclear and cytoplasmic proteins was performed as reported previously [19], [20]. Immunoprecipitations were performed using 600 µg of total cell lysate in HNTG buffer (20 mM HEPES, 150 mM NaCl, 10% glycerol, 0.1% Triton X-100) or in PBS-0,2% NP40 (for stathmin and RhoA immunoprecipitates) plus the specific agarose-conjugated primary antibody (FLAG-M2 Affinity gel) and incubating overnight at 4°C. When primary antibodies were not agarose conjugated, protein A or protein G Sepharose 4 Fast Flow (Amersham Biosciences) was added during the last 2 hours of incubation. Immunoprecipitates were washed six times in HNTG buffer, and then nine parts were resuspended in 3× Laemmli sample buffer with 50 mM dithiothreitol and one part was resuspended in kinase buffer. Kinase assays were performed as previously described [19], [22] using 2 µg of histone H1 as the substrate (Upstate Biotechnology) and [γ-^32^P]ATP (PerkinElmer Life Sciences) to reveal phosphorylation levels. p27 kinase inhibitory activity was also tested with an *in vitro* assay using recombinant cyclin-CDK complexes. Different amounts of recombinant p27 mutants were incubated with 40 ng of recombinant cyclin A_2_-CDK2 complex (SignalChem) or with 300 ng of cyclin D_1_-CDK4 (SignalChem) complex, on ice for 1,5 hour. Then samples were subjected to kinase assay using, respectively, histone H1 or Rb protein as the substrate. For immunoblotting, 40 µg of proteins was separated by 4–20% SDS-PAGE (Criterion precast gel; Bio-Rad) and transferred to nitrocellulose membranes (Hybond C; Amersham Inc.). Membranes were incubated with primary antibodies as indicated and then with horseradish peroxidase-conjugated secondary antibodies (GE Healthcare) for ECL detection (GE Healthcare) or Alexa-conjugated secondary antibodies (Invitrogen) for Odyssey infrared detection (Licor). For immunoprecipitates, rabbit immunoglobulin G and mouse immunoglobulin G True Blot (eBioscience) secondary horseradish peroxidase-conjugated antibodies were used. Quantification of the immunoblots was done using the QuantiONE software (BioRad) or the Odyssey infrared imaging system (Licor Biosciences). Primary antibodies were from: Transduction Laboratories (p27, metablastin/stathmin and CDK2), Santa Cruz (p27 C19, p27 N20, CDK4, vinculin, Skp2), Clontech (Zs green polyclonal antibody), Sigma (Op18/stathmin), Upstate (RhoA), as marker of nuclear extracts we used an anti-HMG2A kindly provided by Prof. G. Manfioletti.

### In vitro transcription and translation

*In vitro* transcription-translation was performed using the TNT T7 Quick Coupled Reticulocyte Lysate System (Promega). p27^wt^ and p27 mutants cDNAs were cloned in the pTNT vector (Promega) and 1 µg of circular plasmid was added directly to TNT Lysate and incubated in a 50 µl reaction for 1.5 hours at 30°C. The reaction mix contained [^35^S] methionine (PerkinElmer Life Sciences) for the detection of translated proteins. Before doing the experiment, 100 ng of each plasmid were loaded into an agarose gel for mass standardization.

### In vitro protein degradation assay

*In vitro* degradation of p27 was carried out essentially as previously described [5]. Briefly, HT1080 cells were transfected with pHA-Ubiquitin vector and lysed 48 hours later in ice-cold double-distilled water. The sample was frozen and thawed 3 times, and then spun down to pellet debris. The supernatant was retrieved and frozen at −80°C. 1 µg of histidine-tagged p27 was incubated at 37°C for different times in 50 µl of degradation mix containing 200 µg of lysate from HT1080 cells, 50 mM Tris-HCl (pH 8.0), 5 mM MgCl_2_, 1 mM DTT and 2 mM ATP. After the indicated times, reactions were stopped by adding 5× Laemmli buffer and loaded onto 4–20% polyacrylamide gel. p27 degradation was analyzed by immunoblotting with an anti-p27 monoclonal antibody.

### RNA Extraction, RT-PCR and Real-Time PCR

Total RNA from transfected cells was extracted using the RNeasy kit (Qiagen). Two micrograms of total RNA was retrotranscripted using AMV Reverse Transcriptase and random hexamers according to the provider's instruction (Promega); 1/20 of the obtained cDNAs were then amplified using the upstream primer for the human p27 sequence and the M13 reverse primer that target pDNR vector, to not amplify endogenous p27 transcript, or with primers for human GAPDH mRNA. Quantitative Real-Time PCR analysis was performed with the same cDNAs using SYBR Green PCR Core Reagents (Applied Biosystems).

### Analyses of cell proliferation

To evaluate cell cycle progression 3T3 p27^KO^ cells were transduced with the indicated mutants and 24 hours after transduction cells were starved and then released in DMEM complete. DNA content was determined by propidium iodide staining using FACS analysis as previously reported [19]. Data were analyzed with a cell cycle analysis software program to calculate the distribution of cells in G1, S and G2/M phases of the cell cycle. For colony assay, 24 hours post-transduction SCC9 cells were trypsinized, counted and seeded at density 1000 and 5000 cell/100 mm dish and incubated in their complete growth medium. Two weeks later plates were stained with crystal violet and colonies counted. Results were expressed as percentage of growth inhibition comparing the colony numbers of each condition to the colony numbers formed in the plate transduced with AdGFP control vector. Experiments were performed in quadruplicate and data represents the mean value of three independent experiments. The transduction efficiency was calculated counting the number of green cells in 10 randomly selected fields, 24 hours after transduction. After 15 days, 60–80 colonies were counted for each mutants and the percentage of green colonies was determined.

### Morphology in 3D

For the evaluation of cellular morphology in 3D matrix, U87MG cells were included in Matrigel (6 mg/ml; BD) 10-µl drops on a coverslip (5000 cells/drop) and maintained for 1 hour upside down at 37°C, to allow Matrigel polymerization. Then, complete medium was added. Drops were incubated for 24 hours and photographed using a phase-contrast microscope to evaluate cell morphology.

### Time-Lapse video microscopy

For the evaluation of cell motility following adhesion to FN cells were transfected with EGFP-p27 plasmids in the presence or not of DsRed-Stathmin vectors and 24 hours later plated on FN coated glass bottom dishes (WillCo Wells BV) that allow the use of the 63× immersion glycerin objective. Cells were then incubated at 37°C in 5% CO_2_ atmosphere in the Leica Time Lapse AF6000LX workstation equipped with the Leica DMI 6000 motorized microscope and an environmental chamber for the proper setting of temperature humidity and CO_2_ concentration. The microscope allows the acquisition of multiple fields on the X, Y and Z axes and is computer assisted. The AF6000 Software (Leica) allows the acquisition of the images on the desiderate frames and periods of time. Images were collected every 2 minutes for 2 hours and used to create a video (15 images per second) and analyzed with a cell tracking software (Leica) to collect different locomotion parameters [21], [24]. In brief, cells were randomly selected and their x/y coordinates tracked using the IM2000 software (Leica), which allows automated or manual cell tracking in order to obtain cell speed (micron/min) and total distance covered (micron) by the cells. At least 3 independent wells/cell type have been analyzed.

### Migration experiments

Migration assays were performed essentially as previously described [19]. Briefly, bottoms of HTS Fluoroblok (Becton Dickinson) were coated overnight at 4°C with 10 µg/ml FN, and then saturated 2 hours at room temperature with PBS 1% BSA. Cells were seeded in the Fluoroblok™ upper chamber and then incubated at 37°C for the indicated times. Migrating cells were evaluated by reading at different time points the lower and the upper sides of the membrane with the Spectrafluor reader (Tecan). Each experiment was performed at least 3 times, in duplicate. Migration was then blocked by fixing the Fluoroblok membranes in 4% PFA to allow the count of migrated cells.

## Results

### p27 protein expression is affected by the T198A point mutation but is not dependent on the T198 phosphorylation

**Figure 1:**
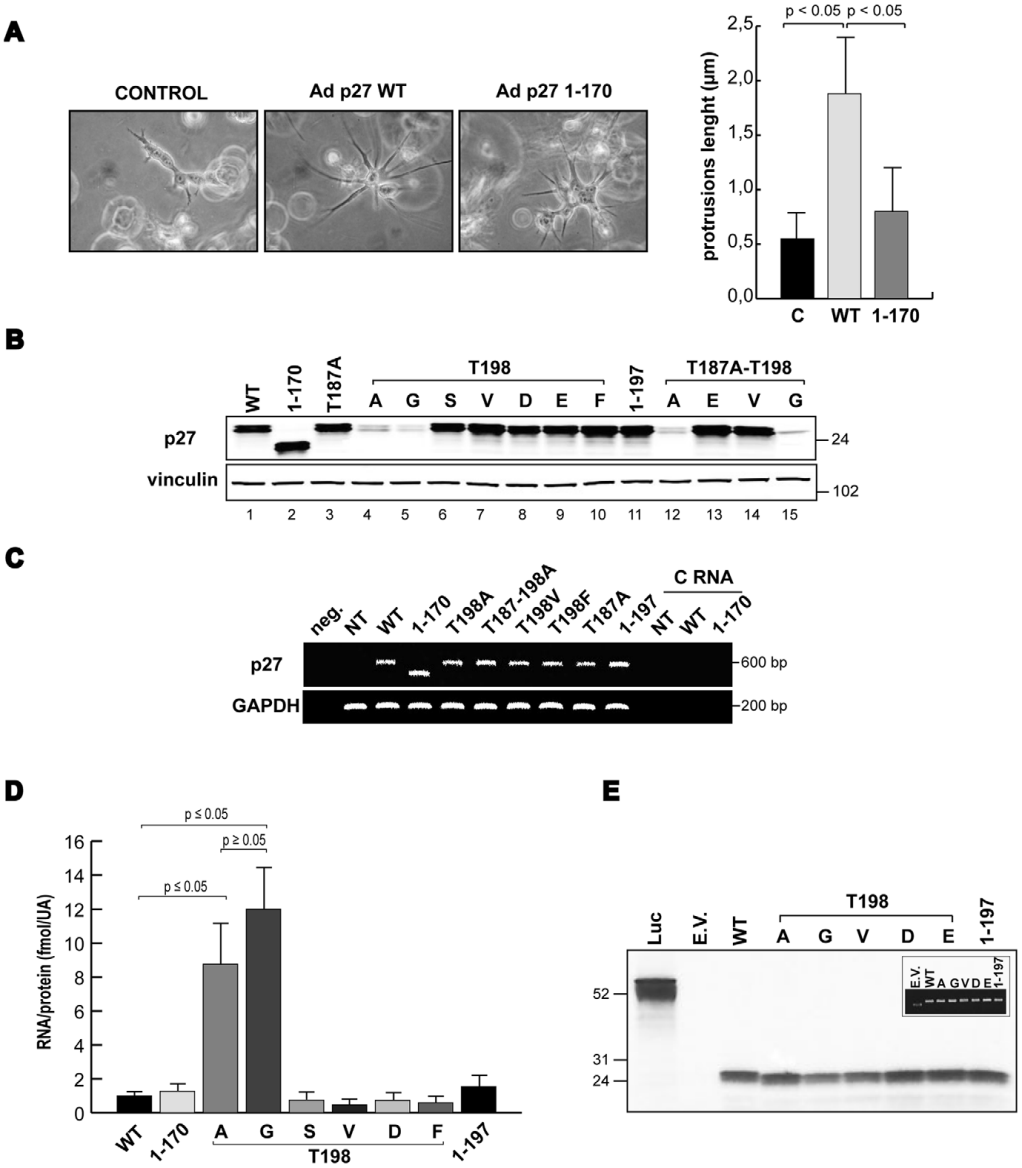
p27 protein expression is affected by the T198A/G point mutation. **A**. U87MG cells were transduced with the indicated Ads, included in 3D Matrigel matrix and images were taken 24 hours later (20× objective). In the graph the length of cell protrusions is reported. Data represents the mean value (± standard deviation) obtained measuring protrusions length in at least 20 cells for each type of transduction in 3 independent experiments. **B**. Western blot analysis of p27 expression in SCC9 cells transiently transfected with different p27 mutants cloned in the pDNR vector and analyzed 48 hours after transfection. Vinculin was used as the loading control. (A = alanine, G = glycine, S = serine, V = valine, D = aspartic acid, E = glutamic acid, F = phenylalanine). **C**. RT-PCR on RNA extracted from SCC9 treated as in B. GAPDH was used as internal control. NT = Non Transfected cells, C RNA = amplification of non retro-transcribed RNA. **D**. SCC9 cells were transfected with pDNR p27^WT^ and with the indicated mutants and 48 hours later RNA and protein extracts were prepared from each plate. Results represent the ratio between the fentomoles of p27-mRNA evaluated by qRT-PCR analysis and the amount of p27 protein expression evaluated by densitometric analysis of the blots and normalized to the loading control vinculin. Data represents the mean value (± standard deviation) of three independent experiments. Statistical analysis was carried out using Student's *t* test. **E**. p27^wt^ and the indicated T198 mutants cDNAs were *in vitro* transcribed and translated, loaded into SDS-PAGE gel and subjected to autoradiography. Inset shows control of plasmidic DNA quantification used in this experiment. Luc = luciferase positive control vector, E.V. = empty vector.

We recently demonstrated that the C-terminal portion of p27 is important for its ability to control MT stability via stathmin interaction and this, in turn, impinge on cell motility [19]–[21], [24]. By studying the effects of p27^WT^ protein on the regulation of glioblastoma cell growth and motility we observed that the WT protein was able to stimulate the formation of neuron like protrusions in U87MG cells (Video S1), thus confirming our previous observation that p27 is able to control protrusion formation in 3D context [24], also in glioblastomas. Further data demonstrated that p27 stimulated protrusion formation in U87MG cells through its C-terminal tail, since the deleted mutant p27^1–170^ was ineffective (Figure 1A). To better understand the mechanism of regulation of cell shape and motility by p27, we decided to generate specific point mutants in those residues comprised among amino-acids 170 and 198 that have been proposed to have a role in the regulation of p27 protein. Indeed, two residues appeared of particular interest in this region, namely the T187, that plays a fundamental role in CDK-mediated degradation of p27 [11] and the T198 which has been proposed to regulate protein stability [18], autophagy [25] and cell motility [17].

First, we generated a p27^T198A^ non-phosphorylable mutant to verify whether or not the modification of p27 last amino-acid had any role in morphological and motility phenotypes of glioblastoma cell lines. Adenoviruses expressing the WT, the 1–170 and the T198A p27 proteins were generated and used to transduce the U87MG cell line. The T198A mutation resulted in a strong abrogation of p27 protein expression (Figure S1). This effect was independent from the phosphorylation of the T187 since it was not rescued by the concomitant mutation of T187 in Alanine and were confirmed using DNA transfection instead of adenoviral transduction using 3 different cell lines (Figure S1 and Figure 1B). Altogether these data confirm that T198 is a key residue for p27 protein stability and its effect is not dependent on the phosphorylation of T187.

**Figure 2:**
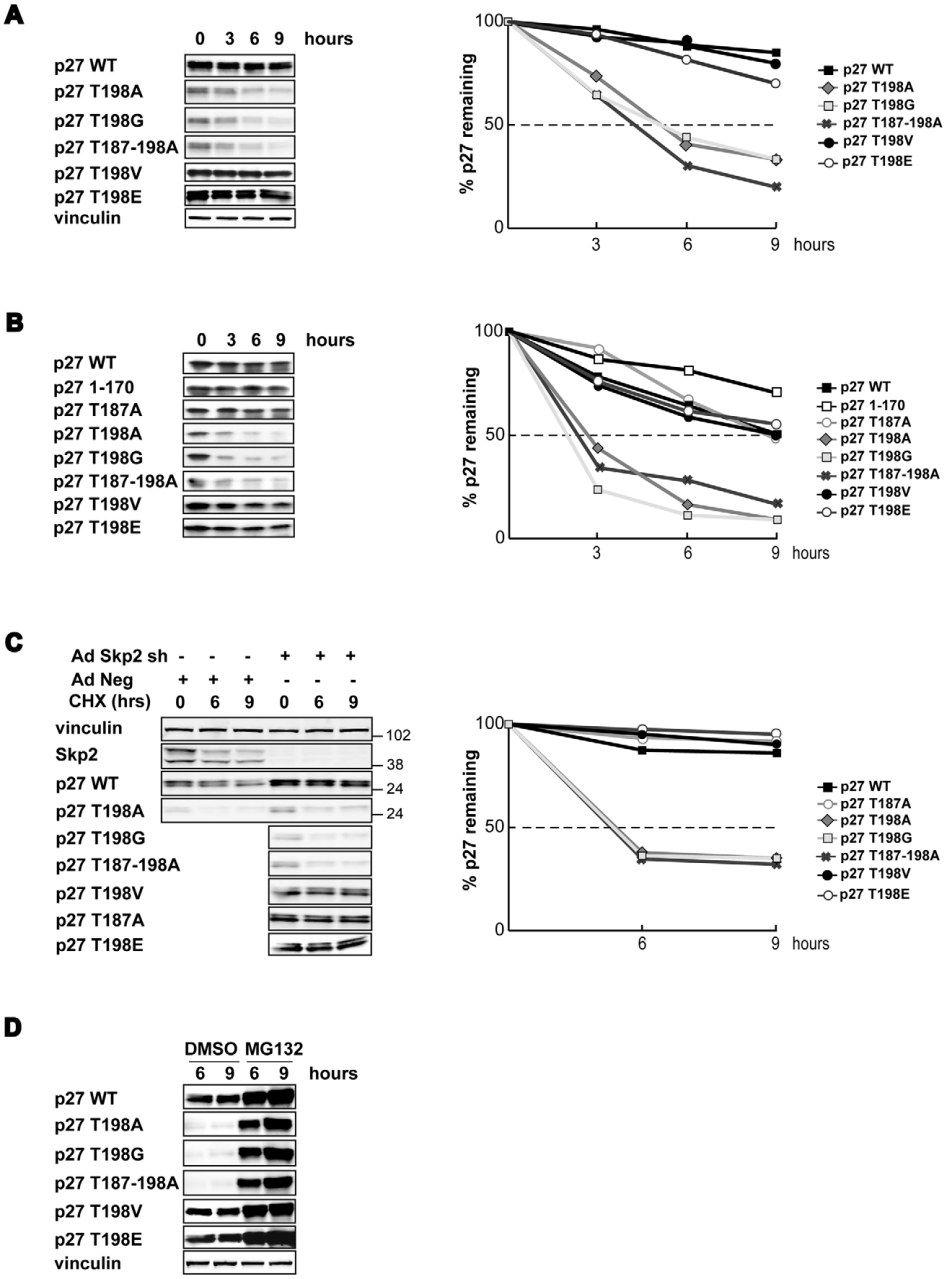
T198A/G substitution markedly decrease p27 protein stability. **A**. Western Blot analysis of p27 expression in SCC9 cells were transfected with the indicated p27 mutants and 48 hours later treated with cycloheximide (CHX) for 3, 6 and 9 hours. The densitometric analysis of the blots is reported in the graph (right) and is expressed as percentage of remaining protein respect to untreated cells. **B**. Western Blot analysis of p27 recombinant proteins in *in vitro* degradation assay using bacterial His-tagged recombinant p27 proteins incubated with proteasome extracts for 3, 6 and 9 hours. Quantification of this degradation assay is reported in the right graph and expressed as described in A. **C**. Western blot analysis of p27 and Skp2 expression in MDAH cells transduced with AdSkp2 or with AdNeg (Control) shRNAs in which the different p27 proteins were expressed and treated or not with CHX for 6 and 9 hours. Total protein extracts were analyzed by western blot using the indicated antibodies (left). The densitometric analysis of a representative Skp2 silencing experiment is reported in the graph (right) and expressed as in A. **D**. Western blot of 3T3 p27^KO^ fibroblasts expressing the different p27 mutants as indicated and treated with MG132 (50 µM) or DMSO for 6 and 9 hours. A typical experiment is shown.

In contrast with this observation several lines of evidence demonstrated that the p27^1–170^ mutant (that lacks both the T198 and the T187 residues) has an expression comparable to that of the WT protein (Figures 1B, 2B, S1) and references [19], [21]. In order to clarify this point the p27^1–197^ mutant was generated in which only the last amino-acid was deleted. As shown in Figure 1, p27^1–197^ was expressed at levels similar to the WT protein indicating that the phosphorylation of p27 on T198 is not required to govern the protein stability (compare lane 1 with lane 11 in Fig. 1B).

We then reasoned that the Threonine homolog non-phosphorylable amino-acid is the Valine and not the Alanine while the Glutamic Acid (E) and Aspartic Acid (D) represent the pseudo-phosphorylated mutation of Threonine and Serine, respectively (Figure S2). In accord with the observation obtained with the deletion p27^1–170^ and p27^1–197^ mutants, both T198V and T198E mutants displayed an expression level similar to that of the WT p27 protein (compare lane 1 with lanes 7 and 9 in Figure 1B). Similarly, the double mutants T187A-T198E and T187A-T198V were expressed as the WT protein (Figure 1B).

Being the Alanine the smallest of the 20 amino-acids after the Glycine, we hypothesized that the steric hindrance of the last amino-acid in p27 rather than the addition of a phosphate group to the T198 was important for the correct expression of the protein. To prove this hypothesis the p27 T198 was changed either in Glutamic Acid (D), Serine (S), Phenylalanine (F) or Glycine (G). All the mutants but T198G displayed level of expression similar to that of the WT protein (Fig. 1B). Consistently, the double mutant T187A-T198G was expressed at very low level when compared to the expression of the WT protein (Figure 1B). Thus, only the substitution of T198 with A or G, the two smallest amino-acids, resulted in the reduced p27 expression, confirming that the size of the last amino-acid rather than the Threonine phosphorylation was important for the regulation of its expression.

### p27^T198A^ displays an increased proteasome-dependent degradation that is not dependent on the T198 phosphorylation

We then tried to understand the mechanism by which the T198A/G substitution could regulate p27 expression. We first looked at the transcription of the different constructs that, as expected, was similar in all mutants as indicated by semiquantitative RT-PCR (Figure 1C). These results were confirmed by quantitative Real Time PCR experiments coupled with western blot analyses in the same transfectants. The results of three independent experiments showed in Figure 1D obtained by comparing the protein expression (in arbitrary units) with the fentomoles of p27 mRNA present in each transfectant, demonstrated that when the T198 in p27 was mutated in G or A only 1/10 of the protein was found per each fentomole of mRNA as compared with the ratio observed for the WT protein (Figure 1D). In any of the other mutants tested we were not able to find any significant difference with the ratio observed for the WT protein.

Using *in vitro* transcription-translation assays we also excluded that the differences in protein expression resided in the ability of the mRNA to be correctly translated in protein since similar amount of protein was produced *in vitro* per each nanogram of plasmid used (Figure 1E).

Collectively these data suggested that the different expression of p27^T198A/G^ mutants could be linked to differences in protein stability. To test this hypothesis we looked at the stability of p27^WT^ and mutant proteins in cells treated with cycloheximide (10 µg/ml) for different times. This experiment clearly showed that the stability of mutants containing either G or A as last amino-acid was lower than that observed for the WT protein, with an half life of about 4,5 hours for the A/G containing mutant respect to about 12 hours for the WT or the non A/G T198 (T198E and T198V) mutants (Figure 2A).

As a complementary approach we utilized recombinant p27 proteins produced in *E. coli* and analyzed them in a proteasome dependent degradation assay. Western blot analysis demonstrated that the degradation rate of p27^T198A/G^ mutants tested in the presence of proteasomal extract derived from HT-1080 cells transfected with HA-tagged ubiquitin was higher respect to that of the WT protein or substituted with the V or E amino-acids (Figure 2B). Quantitative analysis demonstrated that mutants containing p27^T198A/G^ displayed a half-life of about 2.5 hours respect to the 7–9 hours of p27^T198V^, p27^WT^ or p27^T198E^. Also in this case the concomitant mutation of T187 was not able to rescue p27 stability (Figure 2B), demonstrating that the presence of A/G in the last position of p27 was dominant respect to the mutation of T187. Accordingly, the p27^1–170^ mutant that lacks both T198 and T187 resulted even more stable in the same assay (Figure 2B).

These data suggested us that the presence of a small amino-acid in the last residue of p27 protein resulted in increased proteasome dependent degradation and that this process was independent from the phosphorylation of T187. Since phosphorylation of T187 is an event necessary for the Skp2 mediated degradation of p27 [7]–[9], [11], our data pointed to a Skp2 independent pathway leading to p27 down-modulation.

This hypothesis was sustained by subsequent results coming from experiments using MDAH 2774 cells where the Skp2 silencing level was optimal. Use of shRNA directed against Skp2 resulted in increased expression of the p27^WT^, p27^T198V^ or p27^T198E^ proteins but produced only minor effects on the half-life of p27^T198A^ or p27^T198G^ (Figure 2C). However, the experiments confirmed that the low levels of p27^T198A/G^ were due to their enhanced protein degradation since their expression could be rescued by the treatment of the cells with the proteasome inhibitor MG132 (Figure 2D).

### Mutation of T198 in Alanine increased proteasome dependent degradation of p27 independently by its CDK binding ability

**Figure 3:**
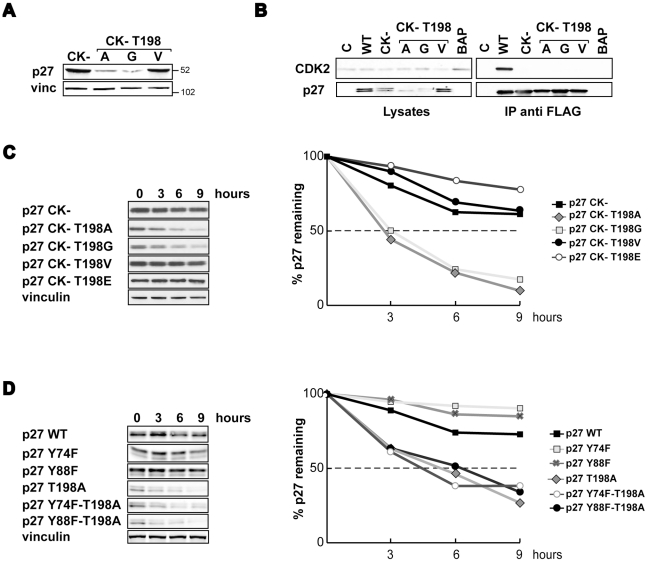
p27^T198A/G^ increased proteasome-dependent degradation is not dependent on CDK-binding. **A**. Western blot analysis of p27 expression in HT1080 transfected with the indicated vectors and analyzed 48 hours after transfection. **B**. Western blot analysis of p27 and CDK2 expression in SCC9 cells transfected with the indicated FLAG-p27 or control (pFLAG-BAP) vectors analyzed 48 hours after trasfection. The amount of CDK2 bound to the different p27 mutants is shown in the right blots (IP anti-FLAG). **C and D**. Western blot analysis of p27 expression in HT1080 transfected with the indicated vectors treated with CHX for 3, 6 and 9 hours. In the graph is reported the densitometric analysis of the blots.

We also tested the possibility that CDK2 binding could be involved in the regulation of p27^T198A/G^ stability. To this aim we generated p27^CK−^ mutants in which the 30–32 and 62–64 amino-acids were mutated and the p27 binding ability to the CDK2/cyclin A/E complexes abolished [26]. The expression levels of p27^CK−^ were similar to those of the WT protein (data not shown). When p27^CK−^ was also mutated in the last amino-acid in either A, G or V, only the p27^CK−/T198V^ mutant was expressed at levels similar to that of the control protein (Figure 3A). Immunoprecipitation experiments demonstrated that as expected the CK− p27 mutants did not bind the CDK2 protein (Figure 3B) and the analysis of protein stability in the presence of cycloheximide demonstrated that the CK− mutation did not alter the stability of p27^T198A/G^ mutants in two different cell lines (Figure 3C and Figure S3).

It has also been proposed that the phosphorylation of Tyrosines 74, 88 and 89 is important for p27 protein degradation and is related to conformational changes in its C-terminal tail. We thus hypothesized that they could be involved in the generation of the phenotype observed with the T198A/G mutants. Preventing the phosphorylation of either Y74 or Y88 did not alter the stability of the T198A mutant although the single substitution in Y74 or in Y88 slightly increased p27 protein stability, as judged by experiments of cycloheximide treatment (Figure 3D). In this setting, the half life of p27^Y74F-T198A^ and p27^Y88F-T198A^ was about 5 hours, thus similar to the p27^T198A^ mutant, while that of the single p27^Y74F^ and p27^Y88F^ was about 15 hours, thus longer than that of the WT protein (12 hours of half-life).

Altogether these data demonstrate that the T198A/G substitution causes an increased degradation of p27 protein but that this effect is not related to the binding with the Cyclin/CDK complexes.

### The lack of p27 phosphorylation on T198 does not affect cancer cell proliferation

The phosphorylation of p27 on T198 has been linked to the control of cell growth and motility [17]–[18]. To verify whether or not this was true also in our model system we compared the effects of the overexpression of the p27^T198A^ mutant with that of p27^WT^, p27^T198E^ and p27^T198V^ proteins on the regulation of both cell proliferation and motility.

To evaluate cell growth we transduced SCC9 squamous cell carcinoma with an adenovirus encoding the GFP protein together with the different p27 mutants, under the control of two different CMV promoters. Using this tool it is possible to follow the transduced cells that express the exogenous protein avoiding the influence of the GFP protein on the studied transgene. Moreover, the expression of the GFP protein is not influenced by the protein stability of the transgene, as occurs with the GFP-fusion proteins.

**Figure 4:**
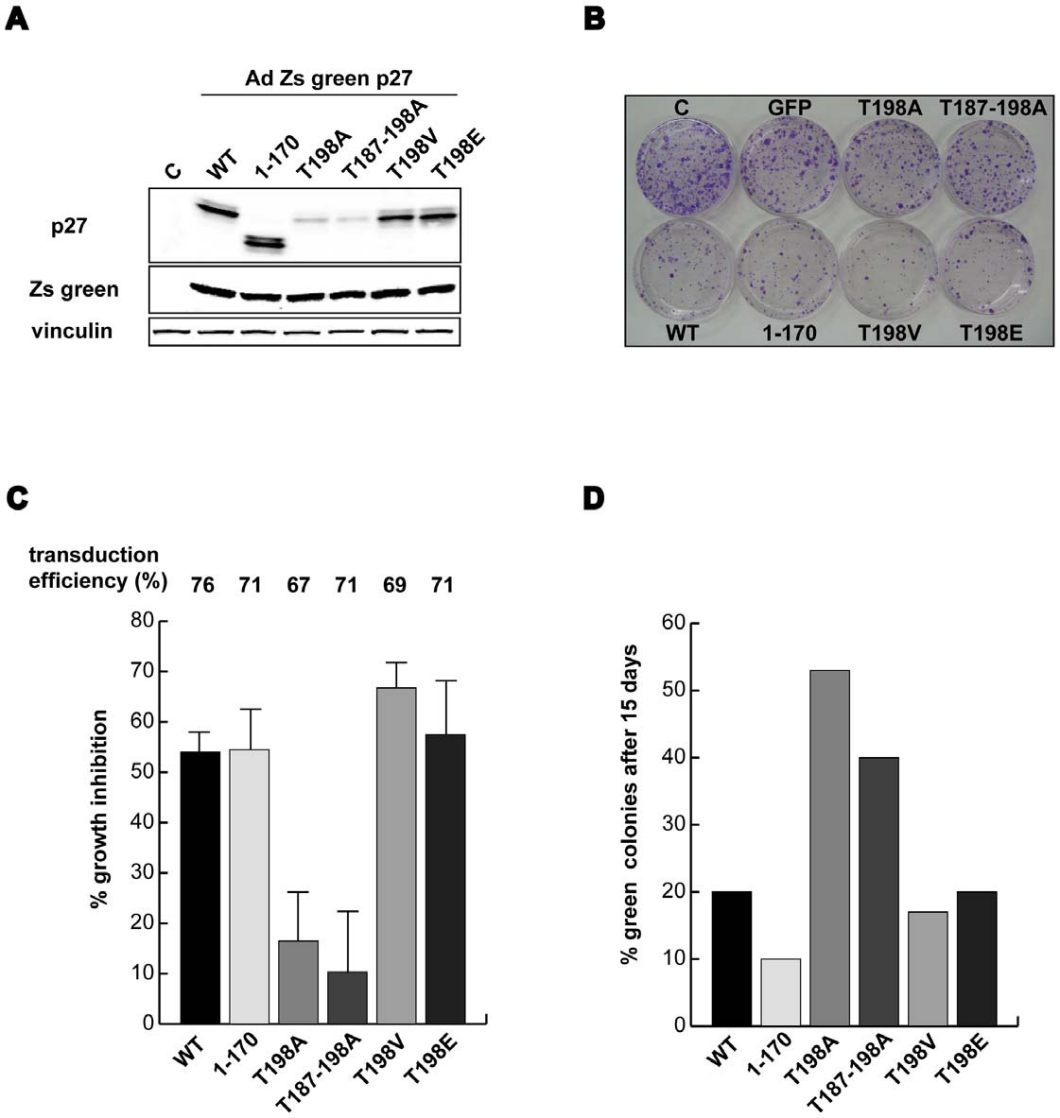
The lack of p27 phosphorylation on T198 does not affect cell cycle progression. **A** Western blot analysis of p27 and Zs green expression in SCC9 cells transduced with the indicated AdZsgreen viruses and analyzed 24 hours post-transduction. **B**. Typical image of colony assay analysis in SCC9 cells transduced as in A and allow to grow for 15 days. **C**. Quantification of growth inhibition induced by the different p27 proteins respect to control cells evaluated by colony assay. Data represents the mean value of three independent experiments performed in quadruplicate. The mean transduction efficiency (% of green cells over the total) is reported. **D**. Percentage of colonies expressing the Zs Green protein 15 days post-transduction as determined counting at least 80 colonies for each mutants. A representative experiment is shown.

Accordingly, we observed similar levels of GFP protein in the transduced cells coupled with high expression of p27^WT^, p27^1–170^, p27^T198E^ and p27^T198V^ proteins while, as expected, with low expression of the T198A mutants (Figure 4A). The colony assay experiment demonstrated that overexpression of p27^WT^, p27^1–170^, p27^T198E^ and p27^T198V^ proteins resulted in about the 60% of growth inhibition (Figure 4B and C) when compared with controls (non-transduced and GFP-transduced cells). This growth inhibition was directly related to the percentage of transduced cells, (69–76% of GFP-positive cells by using the different Adenoviruses) (Figure 4C). The inhibition exerted by the T198A mutants was less than 20%, thus lacking any significant effect on cancer cell growth (Figure 4B and C). The absence of cell growth inhibition by p27^T198A^ (and p27^T187-198A^) was likely due to the low levels of proteins expression, as demonstrated by the count of green colonies in cells transduced with the different Adenoviruses and allowed to grow for 15 days. This experiment showed that while less than 20% of cells expressing the WT protein or the p27^1–170^, p27^T198E^ and p27^T198V^ were green, more than 50% of the colonies in cells expressing the p27^T198A^ protein were GFP-positive (Figure 4D), thus demonstrating that the overexpression of p27 in SCC9 cells was able to inhibit cell growth independently from phosphorylation of T198, but only when expressed over a threshold level.

To further analyze the effects of these mutants overexpression in cell cycle, and being p27 mainly involved in G1/S transition, we studied more in details how T198 substitution affects this event. For this purpose 3T3 p27^KO^ were transduced with p27^WT^, p27^T198E^ and p27^T198V^ serum starved for 24 hours and released in complete medium. Cell cycle reentry was evaluated by FACS analysis and biochemically looking at the expression of cyclin A, used as marker of S phase entry. Data showed that no significant differences could be observed in the ability to enter into the cell cycle among the different mutants analyzed. In fact at 16 hours the percentage of S phase cells was 18%, 17%, and 16% for p27^WT^, p27^T198E^ and p27^T198V^, respectively (Figure S4) coupled with a similar increase in the levels of the S phase marker Cyclin A. Moreover, the levels of p27 expression at the same time points were not affected by the mutation of the T198, confirming the results obtained in exponentially growing cells (Figures 1 and 2).

It has been proposed that the T198A mutation results in a higher inhibition of CDK2 containing complexes [18]. We thus tested whether or not T198A substitution could change the ability of p27 to bind CDK2- or CDK4-containing complexes and whether this effect could be related to the lack of T198 phosphorylation.

**Figure 5:**
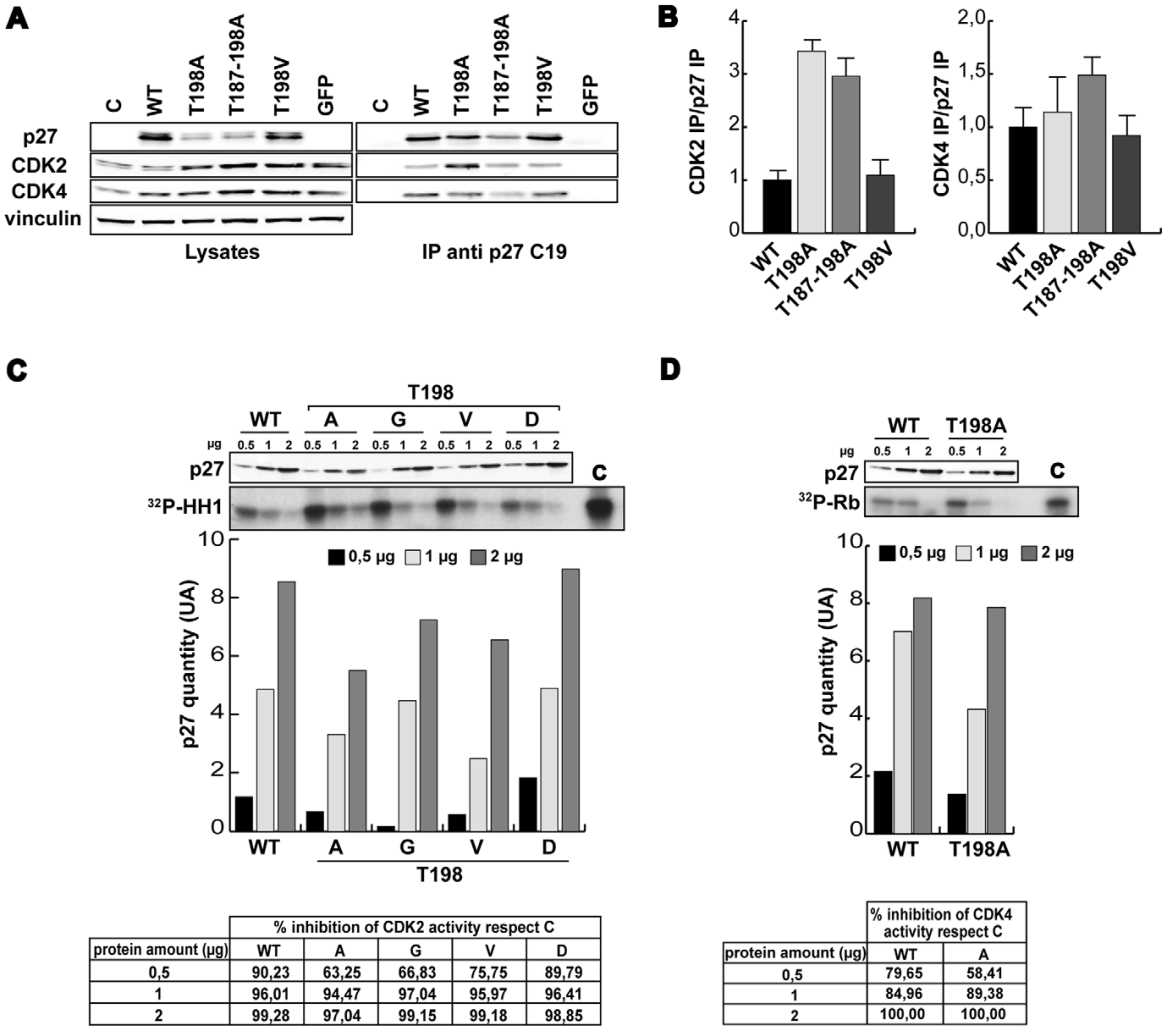
p27^T198A^ and p27^T187-198A^ bind CDK2 with high affinity. **A**. Immunoprecipitation analysis of 3T3 p27^KO^ fibroblasts transduced with the indicated Ad Zs-green p27 and lysed 48 hours post-transduction. IP and lysates were analyzed by western blot using anti-CDK2, anti-CDK4 and anti-p27 antibody. **B**. Quantification of p27/CDK2-4 interaction as evaluated by IP and western blot analysis. Data represents the mean value (± standard deviation) of three independent experiments and are expressed as fold increase respect to the p27^WT^/CDK binding value. **C**. In vitro kinase assay of recombinant cyclin A_2_/CDK2 complex in the presence of increasing amounts (0,5, 1 and 2 µg) of recombinant His-tagged p27^WT^ and mutants proteins as indicated using histone H1 as substrate. In the upper panel the expression of p27 in each reaction is shown. In the lower panel the phosphorylated Histone H1 is shown. A typical assay is reported. In the lower graph the amount of p27 proteins present in each reaction as evaluated by densitometric scanning of the blots is shown. In the lower table the percentage of kinase activity inhibition by the different p27 mutants respect to control kinase activity is reported. **D**. Same as in C using as kinase the recombinant cyclin D_1_/CDK4 complex and pRB fragment as substrate.

To this aim 3T3 p27^KO^ cells have been transduced with vectors encoding for p27^WT^, p27^T198A^, p27^T187-198A^ or p27^T198V^ and the exogenous proteins immunoprecipitated (Figure 5A). The amount of CDK2 and CDK4 bound to the different mutants was then evaluated and normalized respect to the amount of the immunoprecipitated protein (Figure 5B). Results show that T198A protein displays a 3 fold higher affinity for CDK2 respect to the WT one (p<0.01), while the affinity for CDK4 was similar for all mutants (p>0.05) (Figure 5B), thus confirming the results obtained from others [18]. However, an *in vitro* assay performed using either CyclinA_2_/CDK2 or Cyclin D_1_/CDK4 recombinant complexes, demonstrated that the ability of p27^WT^, p27^T198A^, p27^T198G^, p27^T198D^ or p27^T198V^ recombinant proteins to inhibit the CDKs kinase activity was comparable in all mutants (Figure 5C). These data demonstrated that although the T198A mutation increased the ability of p27 to bind the CDK2 containing complexes it did not alter its ability to inhibit the kinase activity and that the effects observed with the T198A mutation cannot be related to the lack of T198 phosphorylation since the T198V and T198D mutants displayed an activity similar to that of the WT protein.

### The T198 phosphorylation in p27 regulates cells motility

**Figure 6:**
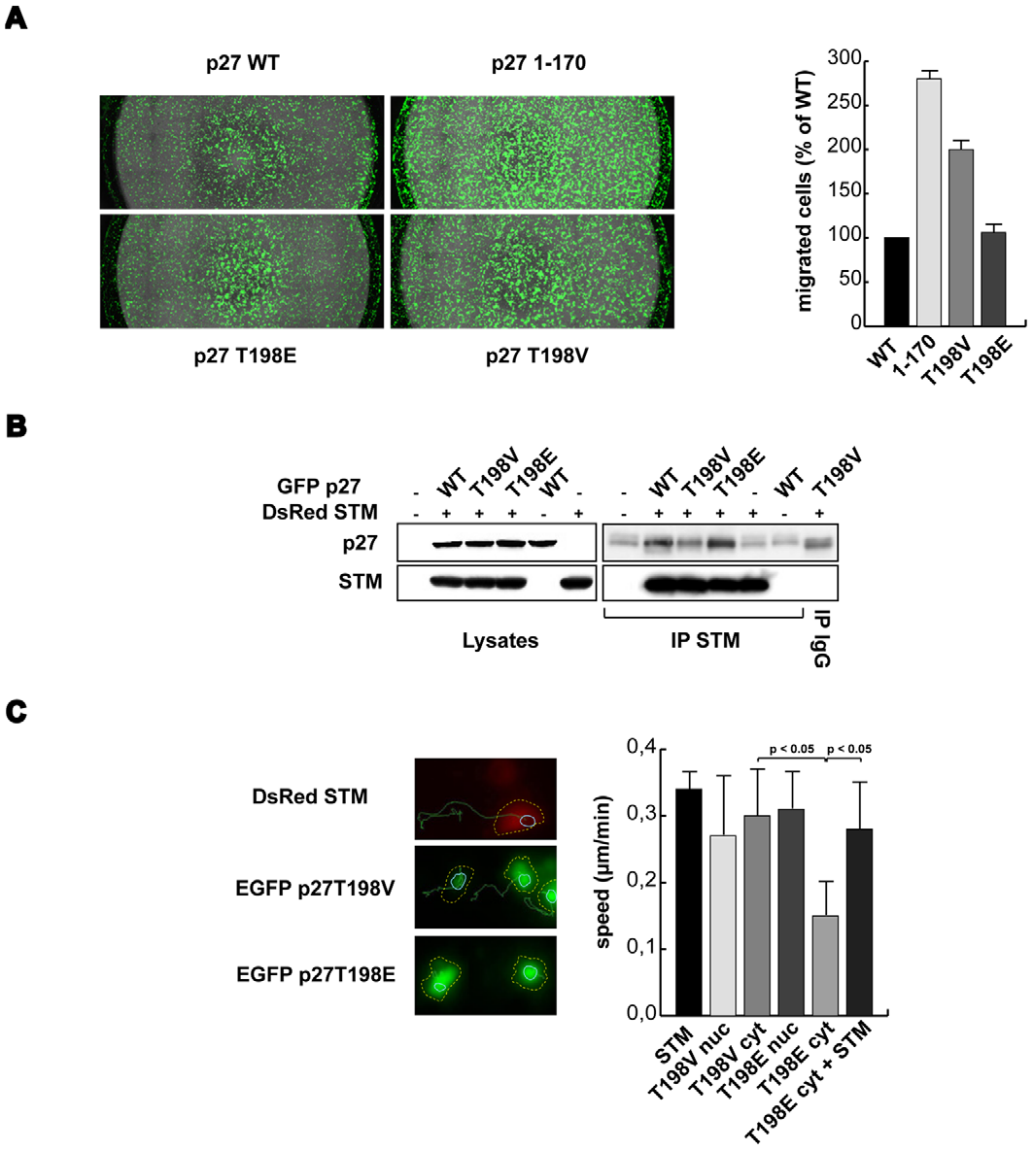
p27 T198 phosphorylation participate in the regulation of cell motility by p27. **A**. HT1080 cells were transduced with the indicated Ad Zs-green p27s and 24 hours later allowed to migrate on FN-coated Fluoroblok. A typical image of Fluoroblok bottom (Migrated cells) is shown. In the graph the quantification of migrated cells is reported. Results are expressed as fold induction respect to control cells. **B**. IP analysis on cytoplasmic fractions of HT1080 expressing pEGFP-p27 mutants and DsRed-stathmin as indicated and adhered on FN for one hour. Cytosolic lysates and IP proteins were then evaluated by western blot analysis using anti-p27 and anti-Stathmin antibodies. **C**. Orthotopical projection of cell paths of U87MG cells transfected with DsRed-Stathmin, EGFP-p27^T198V^ and EGFP-p27^T198E^ allowed to adhere on FN and followed for two hours (left). In the figures yellow dashed lines delimitate the cytoplasm and blue lines the nucleus positions at time 0. In the graph (right) the speed of the transfected cells is reported and represents the mean (±SD) of three independent experiments in which at least 10 cells/experiment were tracked. Statistical analysis was carried out using Mann-Whitney U-Test.

In the last years, a role in the regulation of cell motility has been proposed for p27. In particular, some studies reported a role for T198 phosphorylation in cell motility [17]. To avoid any possible misinterpretation due to the increased proteasomal degradation of p27^T198A^, we compared the activity of p27^T198E^ and p27^T198V^ that have a similar expression and a similar half-life in our cell lines (Figures 1, 2, 3). We thus tested whether or not p27^WT^, p27^T198E^ or p27^T198V^ proteins had any effect on the ECM-driven motility using different experimental settings. We already demonstrated that p27 is able to inhibit the motility of several human cancer cell lines, including HT-1080 fibrosarcoma cells (Figure 6) and [19] and U87MG [data not shown and Ref 20] in transwell migration assays. As already reported, the p27^1–170^ deletion mutant did not affect the ability of these cells to respond to haptotactic stimuli (Figure 6) and [19]–[21]. The use of p27^1–170^, p27^T198E^ or p27^T198V^ revealed that the phosphorylation of T198 plays a role in the regulation ECM-driven motility. As shown in Figure 6A both p27^1–170^ and p27^T198V^ were unable to properly inhibit HT-1080 and U87MG cell migration through a FN-coated transwell while the pseudo-phosphorylated mutant p27^T198E^ displayed an activity similar to the WT protein (Figure 6 and data not shown). To confirm these results we used video time-lapse microscopy to track the motility of p27-EGFP-fusion proteins expressing cells. In these conditions we have been able to look also at the subcellular localization of the transgene, during the migration. Thus, cells were transfected with EGFP-p27^T198E^ or -p27^T198V^ and 1 day later plated on FN-coated dishes and followed for 2 hours using a fluorescence-based time lapse analysis. Strikingly, video time lapse coupled with cell tracking analyses demonstrated that the expression of EGFP-p27^T198V^ both into the nucleus and into the cytoplasm did not interfere with the ability of U87MG cells to properly move on FN (Video S2 and Figure 6C). On the contrary, the expression of the T198E mutant in the cytoplasm (Video S3 and Figure 6C) but not in the nucleus (Video S4 and Figure 6C) almost completely blocked cell motility.

We also verified whether T198 phosphorylation may affect cell motility by favoring p27 nucleo-cytoplasm shuttling [17]. However, in our model system both time-lapse video-microscopy and western blot analyses did not confirm a primary role of T198 phosphorylation in the regulation of p27 subcellular localization following cell adhesion (Figure S5).

The ability of p27 to modulate cell migration has been previously ascribed by its ability to bind other proteins more directly implicated in the regulation of cellular motility, namely RhoA and stathmin [17], [19]–[21]. First we focused on the possible binding of p27 with the small GTPase RhoA, that has been already reported to be influenced by the T198 phosphorylation [17]. To this purpose, we overexpressed in HT-1080 cells both GFP-RhoA and flag-tagged p27^WT^, p27^1–170^, p27^T198E^ or p27^T198V^ proteins and adhered them to FN for 1 hour. No detectable direct association between the two proteins was appreciable in our system (Figure S6A). Similarly, in cells adhered to FN for 1 hour we did not observe the binding of endogenous p27 and RhoA proteins (data not shown). It has been demonstrated that p27 C-terminal tail binds also the MT-destabilizing protein stathmin [19]–[20]. We thus speculated that T198 phosphorylation may favor this interaction in cells adhered to FN. Cells expressing p27^T198E^ or p27^T198V^ proteins, or the p27^1–170^ deletion mutant both as untagged or EGFP-tagged proteins, were co-transfected with FLAG- or DS-Red-tagged stathmin vectors and then adhered to FN for 1 hour. Protein extracts have then been immunoprecipitated using either the anti-p27 (Figure S6B) or the anti-stathmin (Figure 6B) antibodies. Data unveiled that while the association between p27^T198E^ and stathmin was readily detectable (Figure 6B and Figure S6B) the T198V mutation strongly impaired this association (Figure 6B and Figure S6B). As expected and already reported [19]–[20], the deletion of the last 28 amino-acids prevented it (Figure S6B). Importantly, stathmin/p27 interaction was observed only in the cytosolic (Figure 6B) and not in the nuclear fraction (data not shown). To further prove that the interaction between p27 and stathmin plays a role in the control of adhesion dependent cell motility and that this interaction is at least in part regulated by T198 phosphorylation we co-expressed in U87MG cells the DsRed-stathmin and the EGFP-p27^T198E^ protein. Cells were then allowed to adhere to FN coated plates and followed by video-time lapse microscopy. As shown in Figure 6C stathmin expression completely abrogated the ability of cytoplasmic p27^T198E^ to inhibit cell motility (Figure 6C and Video S5). Moreover, the two proteins co-localize in the cytoplasm in response to cell-FN interaction as evaluated by time-lapse confocal video-microscopy (Video S6).

Altogether these data demonstrated that T198 phosphorylation is an important event that may favor the p27/stathmin interaction eventually influencing ECM-driven cell motility.

## Discussion

Here, we investigate the role of T198 phosphorylation in cell growth and motility demonstrating that this event is important in the regulation of cell motility while it does not affect cell proliferation. This observation is in accord with previous studies demonstrating that the C-terminus of p27 is involved in the control of migration but not of cell cycle progression [19]–[21]. We also demonstrate that p27 T198 phosphorylation favors the interaction between p27 and stathmin following cell adhesion to ECM, thus suggesting the mechanism by which the T198 modification could affect cell motility. These data are in agreement with our [23] and others' [27], [28] observations, showing that stathmin activity plays a pivotal role in the regulation of cell migration and metastasis formation. The role of stathmin in cell motility is particularly evident in *in vivo* and in three dimensional settings [23], , which probably represent the experimental situation in which cell plasticity is of particular importance.

We also provide evidence that in this experimental setting the p27/RhoA interaction is not implicated since no direct binding between the two proteins was detectable, even after overexpression of both proteins (Figure S6A). However, we recently demonstrated that the interaction between p27 and stathmin indirectly affects RhoA activity in mouse fibroblasts. In the absence of p27, increased MT-dynamics results in increased RhoA activity, following cell adhesion to ECM [24]. We cannot exclude that this mechanism works also in this system and that RhoA activity participates to the motile phenotype observed in the cells expressing the different p27 mutants. It is to note that the inhibition of the Rho-Rock1 activity results in the formation of cell protrusion in several model system [24], [29]–[31], and we demonstrate here how overexpression of p27 induced protrusion formation in U87MG included in 3D matrices and/or adhered to ECM substrates (Figure 1A and Video S1). This event is dependent on the presence of the last 28 amino-acids and partially on the phosphorylation of T198. In fact we noticed some differences between the activity of p27^1–170^ deletion mutant and the p27^T198V^ point mutant in the regulation of cell motility and protrusion formation and also in stathmin binding capacity. These observations suggest that other residues are important in the regulation of cell motility within the C-terminal portion of p27 and future work will better define the modifications required for proper regulation of cell motility. The relevance of the p27-stathmin-MT dynamics-Rho axis we recently highlighted [24] in the generation of cell protrusion is confirmed by several experimental evidences that demonstrate how p27 [32]–[33] and stathmin [34]–[35] participate in the neurite protrusion formation and in the regulation of neuronal migration, at least in part by modulating Rho small GTPase activity [33], [34]. Our study also suggest that phosphorylation of the last aminoacid did not influence p27 protein subcellular localization. This observation was obtained either by differential extraction of nuclear and cytosolic proteins (Figure S5) and by video time lapse microscopy demonstrating that the frequency of nucleo-cytoplasmic shuttling following cell adhesion to ECM was similar for cells expressing p27^T198V^ or p27^T198E^ (data not show).

On the other hand, our results suggest that phosphorylation of T198 has no effect on cell proliferation and p27 protein stability. These conclusions were drawn by the use of several point mutants and by a number of different approaches that demonstrated how the pT198 does not participate neither in the regulation of p27 proteasomal degradation, nor in its ability to bind the Cyclin-CDK complexes and not even in the regulation of cancer cell growth. These data are only apparently in contrast with some previous publication [18]. We in fact confirmed that the T198A substitution results in increased protein degradation, yet this is not dependent on the T187 phosphorylation and it is not dependent on the binding with the Skp2 protein, as previously suggested [18]. Our data support the possibility that the effects observed with the p27^T198A^ mutant do not reflect a lack of phosphorylation but the presence of a small residue in the last position of p27 protein that may alter the natural unstable and unstructured conformation of p27 C-terminal tail [14], [36]. This conclusion is sustained by several observations and by the use of several mutants differing one each other only for the residue chosen for the substitution of T198 (Figures 1, 2, 3), and it was independently reached by others who recently demonstrated how a protein with a premature stop codon at amino-acid 177 (that results in the presence as last aminoacid of D176) did not display any defect in protein stability when compared to the WT protein [37]. Accordingly, by increasing the size of the last amino-acid from Glycine to Phenylalanine, that contains a big aromatic ring in its structure, the stability of the proteins increased. Moreover, the less stable protein resulted the T-G substitution followed by the T-A and T-V point mutations, which clearly indicated that the steric hindrance of the last amino-acid is a key issue. Vice versa the T198E/D substitutions that mimic the phosphorylation event, did not confer any increased stability to p27 protein both *in vitro* and *in vivo*. Thus it is not the lack of T198 or the lack of its phosphorylation that is responsible for higher protein degradation but only the conformation assumed by p27 when a small amino-acid, such as Alanine or Glycine is present at the very end of the unstructured tail. We can hypothesize that the T198A/G substitution results in an unstable conformation of the C-terminal tail of p27, which is then more rapidly recognized and degraded via the unfolded protein degradation system. Accordingly, it has been demonstrated that, under particular stress conditions, p27 is recognized by the heat-shock protein Hsp27 and this interaction eventually results in accelerated protein degradation [38]. It is interesting to note that in some cancer cell lines p27 C-terminus is cleaved by protease activity [20], [39]–[41] and that this event contributes to control p27 expression levels. Thus, it is possible that such cleavages may result in the exposition of a small aminoacid and then in a more rapid degradation of p27 protein. Mass spectrometry analyses of p27 protein in human tumors could provide a more direct evidence of this hypothesis.

Our work provides new unexpected results on the role of T198 in modulating the functions of p27, and also raises concerns on the correct interpretation of the results coming from studies focused on phosphorylation of threonine and/or Serine only by the use of T/A or S/A substitution.

In conclusion, we show here that the complex regulation of p27 functions include not only the phosphorylation of Threonine 198 but also the three dimensional conformation of its unstable C-terminal tail and how these events eventually play a role in the control of ECM-driven cell motility.

## Supporting Information

Figure S1 **A**. Western blot analysis of p27 expression in U87MG cells co-transduced with AdTRE p27/AdTet-ON 72 hours post-transduction. Vinculin was used for loading control. **B**. Western blot analysis of p27 expression in HT1080 cells transiently transfected with different pEGFP-p27 mutants. **C**, Time course analysis of p27 expression in SCC9 cells co-transduced with AdTRE p27s/AdTet-ON (MOI 80∶40 and 160∶80) as indicated. The expression of p27 and vinculin (loading control) evaluated by western blot analysis is reported.
(TIF)

Figure S2 Comparison of Amino-acids lateral chains. As shown by the structural formula, Valine and Glutamic Acid are, respectively, the non-phosphorylable and phosphomimetic homolog of Threonine, while Alanine and Aspartic Acid are those of Serine.
(TIF)

Figure S3 Western blot analysis of p27 expression in HEK 293 cells transfected with the indicated CK- mutants vectors and treated with CHX for 3, 6 and 9 hours. The densitometric analysis of the blots is reported in the graph (right) and is expressed as percentage of remaining protein respect to untreated cells.
(TIF)

Figure S4 3T3 p27^KO^ fibroblasts were transduced with the indicated Ad Zsgreen p27. 24 hours later cells were serum starved in DMEM 0,1% BSA for 24 hours and then released in complete medium. At the indicated time points cells were fixed and analyzed by flow cytometry for their DNA content and total cell protein extracts were prepared. Cyclin A and p27 levels are reported in the upper panels as evaluated by western blot analyses. Vinculin was used as loading control. The percentage of cells in G1, S and G2/M phases of the cell cycle at each time point is reported in the lower tables. Data represents the mean of three independent experiments.
(TIF)

Figure S5 **A**. Western blot analysis of p27 expression in nuclear/cytoplasmic extracts of HT1080 cells transfected with the indicated FLAG-p27 mutants and adhered 48 hours later on fibronectin for 1 hour. Vinculin was used as control for cytoplasmic fractions and HMG2A as controls for nuclear fractions. **B**. Evaluation of EGFP-p27 localization in U87MG cells transfected with the indicated pEGFP-p27 vectors and adhered to FN for two hours. Data are expressed as percentage of cells with nuclear or cytoplasmic localization.
(TIF)

Figure S6 **A**. Immunoprecipitation (IP) analysis of HT1080 expressing FLAG-p27 and EGFP-RhoA proteins as indicated and adhered on FN for one hour. Total cell lysates and IP proteins were then evaluated by western blot analysis using anti-p27 and anti-Rho antibodies. IPs were performed using both an anti-GFP antibody and the FLAG-M2 affinity gel antibody. No co-precipitation was observed in both the conditions. **B**. IP analysis of HT1080 expressing p27 and FLAG-stathmin proteins as indicated and adhered on FN for one hour. Total cell lysates and IP proteins were then evaluated by western blot analysis using anti-p27 and anti-Stathmin antibodies.
(TIF)

Video S1 Video time-lapse of U87MG cells transfected with EGFP p27^WT^ and adhered on FN for 2 hours. p27^WT^ was able to stimulate the formation of neuron-like protrusions and localizes in the protrusions.
(MOV)

Video S2 Video time-lapse of U87MG cells transfected with EGFP p27^T198V^ and adhered on FN for 2 hours. Expression of p27^T198V^ both in the nucleus and in the cytoplasm did not interfere with the ability of U87MG cells to properly move on FN.
(MOV)

Video S3 Video time-lapse of U87MG cells transfected with EGFP p27^T198E^ and adhered on FN for 2 hours. Expression of the T198E mutant into the cytoplasm almost completely blocked cell motility.
(MOV)

Video S4 Video time-lapse of U87MG cells transfected with EGFP p27^T198E^ and adhered on FN for 2 hours. Expression of the T198E mutant into the nucleus did not interfere with U87MG motility.
(MOV)

Video S5 Video time-lapse of U87MG cells cotransfected with EGFP p27^T198E^ and with DsRed Stathmin and adhered on FN for 2 hours. Stathmin expression completely abrogated the ability of cytoplasmic p27^T198E^ to inhibit cell motility.
(MOV)

Video S6 Time lapse confocal video microscopy of U87MG co-transfected with EGFP p27^T198E^ and with DsRed Stathmin in adhesion on FN for 2 hs. Yellow dots represented the colocalization of p27^T198E^ (green) and stathmin (red) in response to cell-FN interaction. Cells expressing only stathmin (red) or p27 (green) are also present in the field. A single confocal plane was taken during the experim.
(MOV)
