# Supplementary figures and images for: Role of T198 Modification in the Regulation of p27Kip1 Protein Stability and Function
Source: PLoS One. 2011 Mar 14;6(3):e17673. doi: 10.1371/journal.pone.0017673 (PMC3056717; doi:10.1371/journal.pone.0017673)

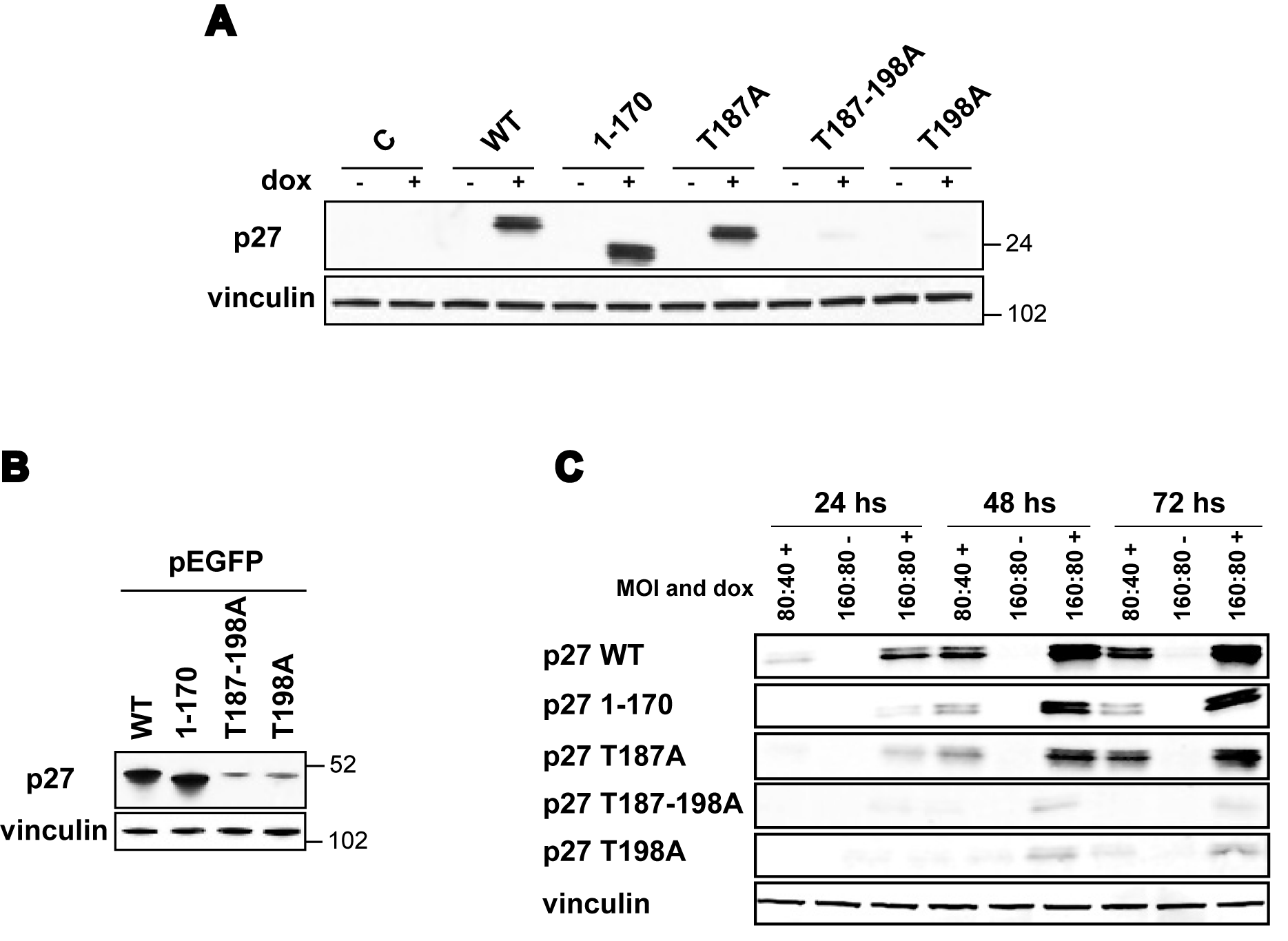

Supplement: Figure S1 — A. Western blot analysis of p27 expression in U87MG cells co-transduced with AdTRE p27/AdTet-ON 72 hours post-transduction. Vinculin was used for loading control. B. Western blot analysis of p27 expression in HT1080 cells transiently transfected with different pEGFP-p27 mutants. C, Time course analysis of p27 expression in SCC9 cells co-transduced with AdTRE p27s/AdTet-ON (MOI 80∶40 and 160∶80) as indicated. The expression of p27 and vinculin (loading control) evaluated by western blot analysis is reported. (TIF) [file pone.0017673.s001.tif]

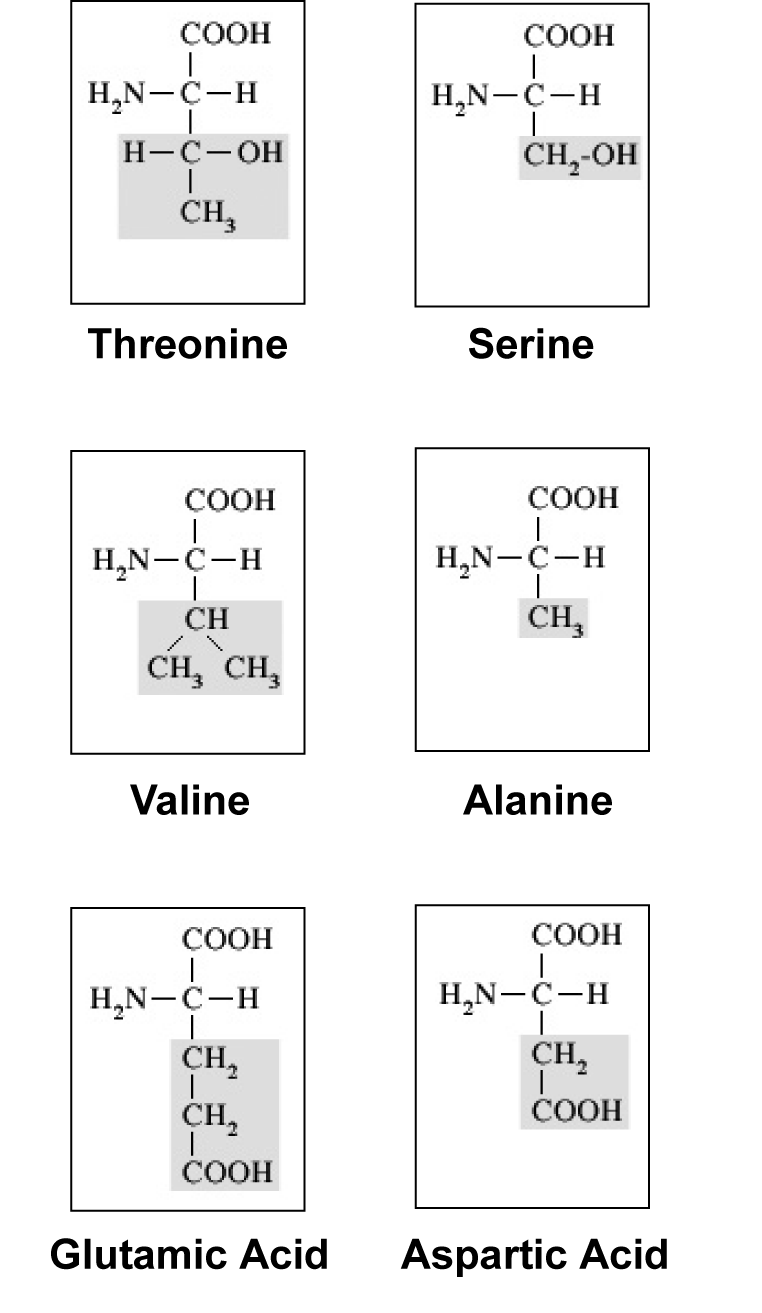

Supplement: Figure S2 — Comparison of Amino-acids lateral chains. As shown by the structural formula, Valine and Glutamic Acid are, respectively, the non-phosphorylable and phosphomimetic homolog of Threonine, while Alanine and Aspartic Acid are those of Serine. (TIF) [file pone.0017673.s002.tif]

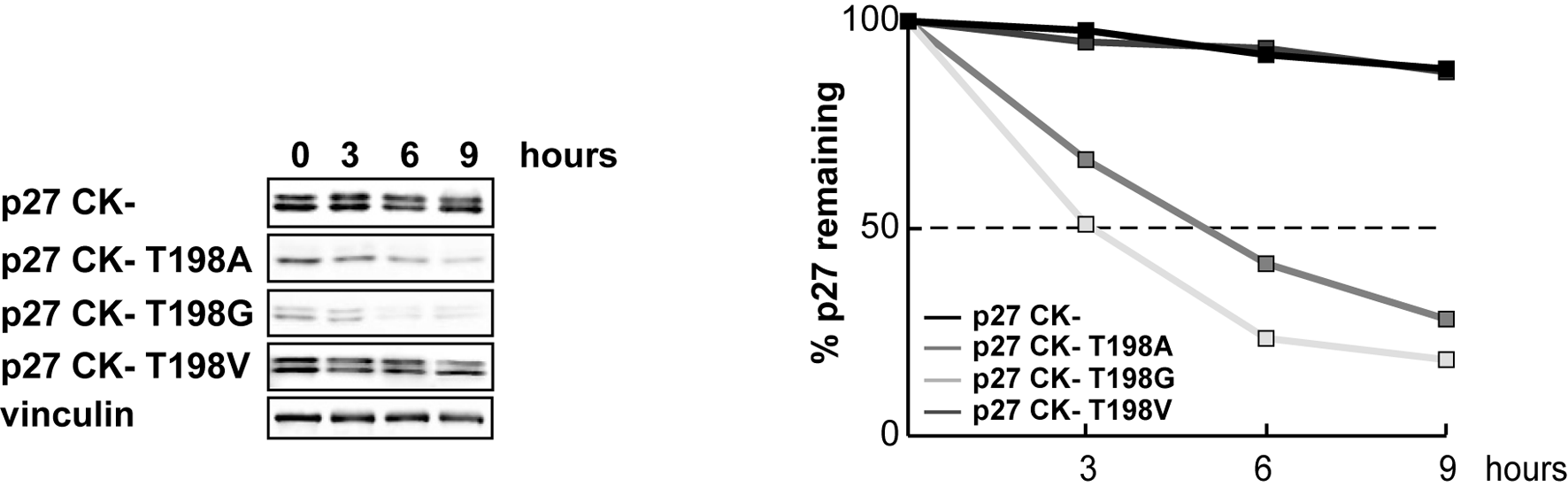

Supplement: Figure S3 — Western blot analysis of p27 expression in HEK 293 cells transfected with the indicated CK- mutants vectors and treated with CHX for 3, 6 and 9 hours. The densitometric analysis of the blots is reported in the graph (right) and is expressed as percentage of remaining protein respect to untreated cells. (TIF) [file pone.0017673.s003.tif]

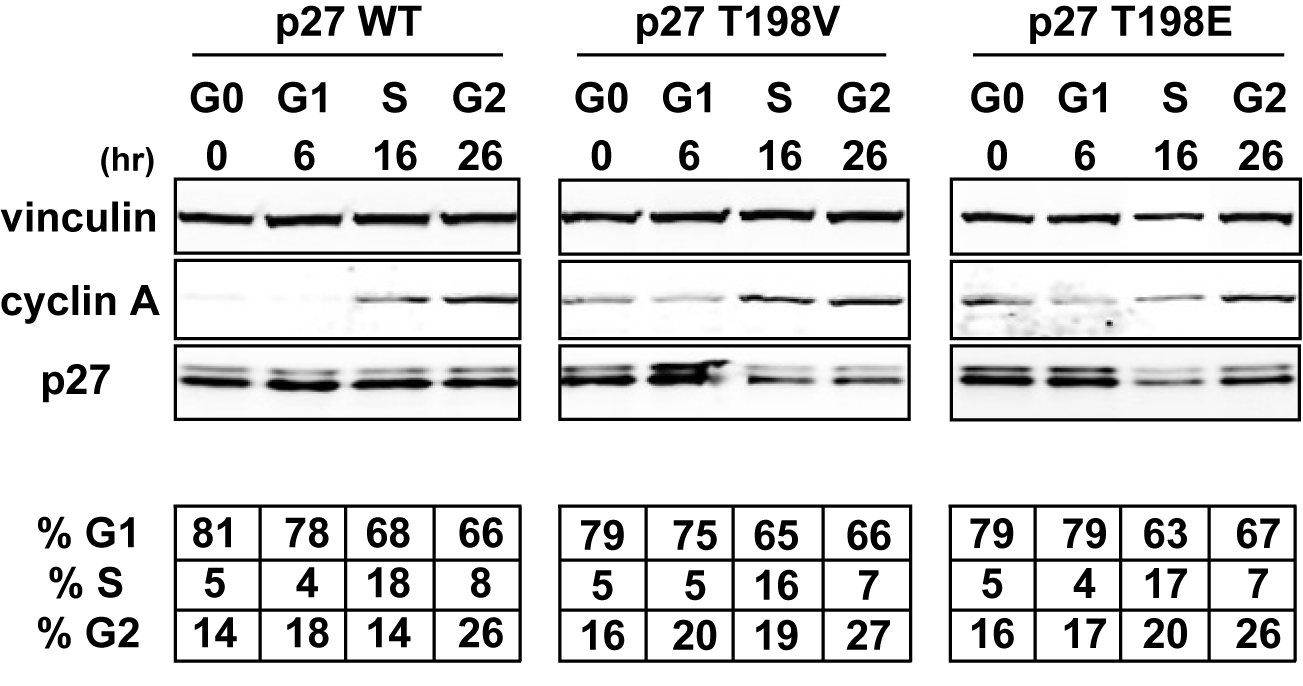

Supplement: Figure S4 — 3T3 p27KO fibroblasts were transduced with the indicated Ad Zsgreen p27. 24 hours later cells were serum starved in DMEM 0,1% BSA for 24 hours and then released in complete medium. At the indicated time points cells were fixed and analyzed by flow cytometry for their DNA content and total cell protein extracts were prepared. Cyclin A and p27 levels are reported in the upper panels as evaluated by western blot analyses. Vinculin was used as loading control. The percentage of cells in G1, S and G2/M phases of the cell cycle at each time point is reported in the lower tables. Data represents the mean of three independent experiments. (TIF) [file pone.0017673.s004.tif]

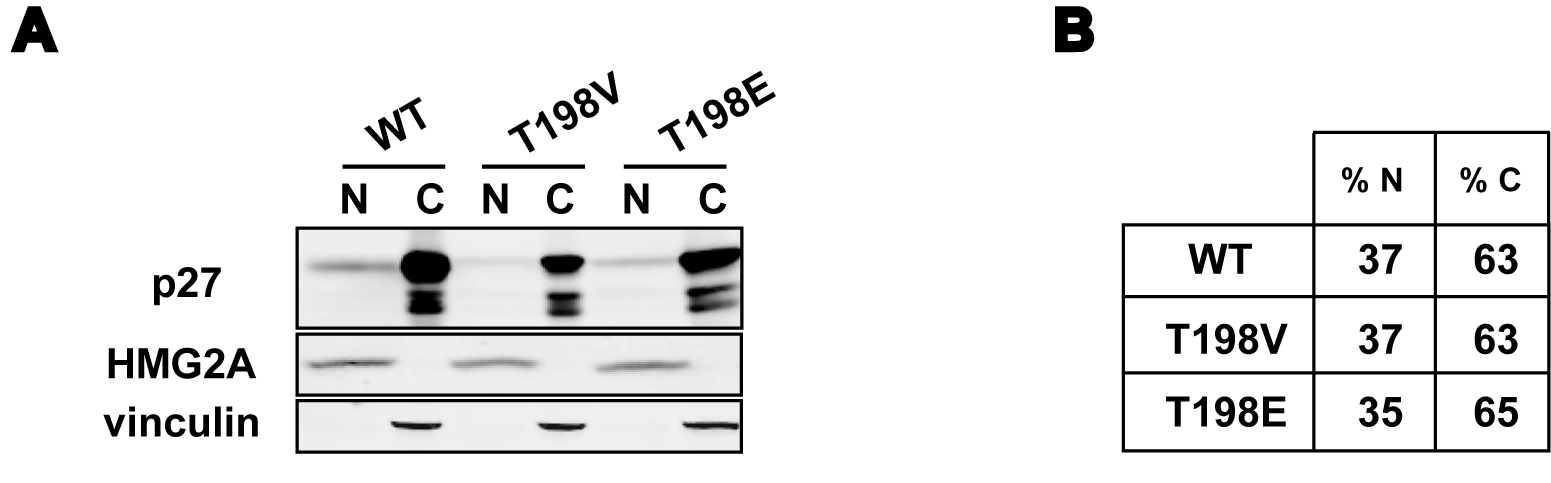

Supplement: Figure S5 — A. Western blot analysis of p27 expression in nuclear/cytoplasmic extracts of HT1080 cells transfected with the indicated FLAG-p27 mutants and adhered 48 hours later on fibronectin for 1 hour. Vinculin was used as control for cytoplasmic fractions and HMG2A as controls for nuclear fractions. B. Evaluation of EGFP-p27 localization in U87MG cells transfected with the indicated pEGFP-p27 vectors and adhered to FN for two hours. Data are expressed as percentage of cells with nuclear or cytoplasmic localization. (TIF) [file pone.0017673.s005.tif]

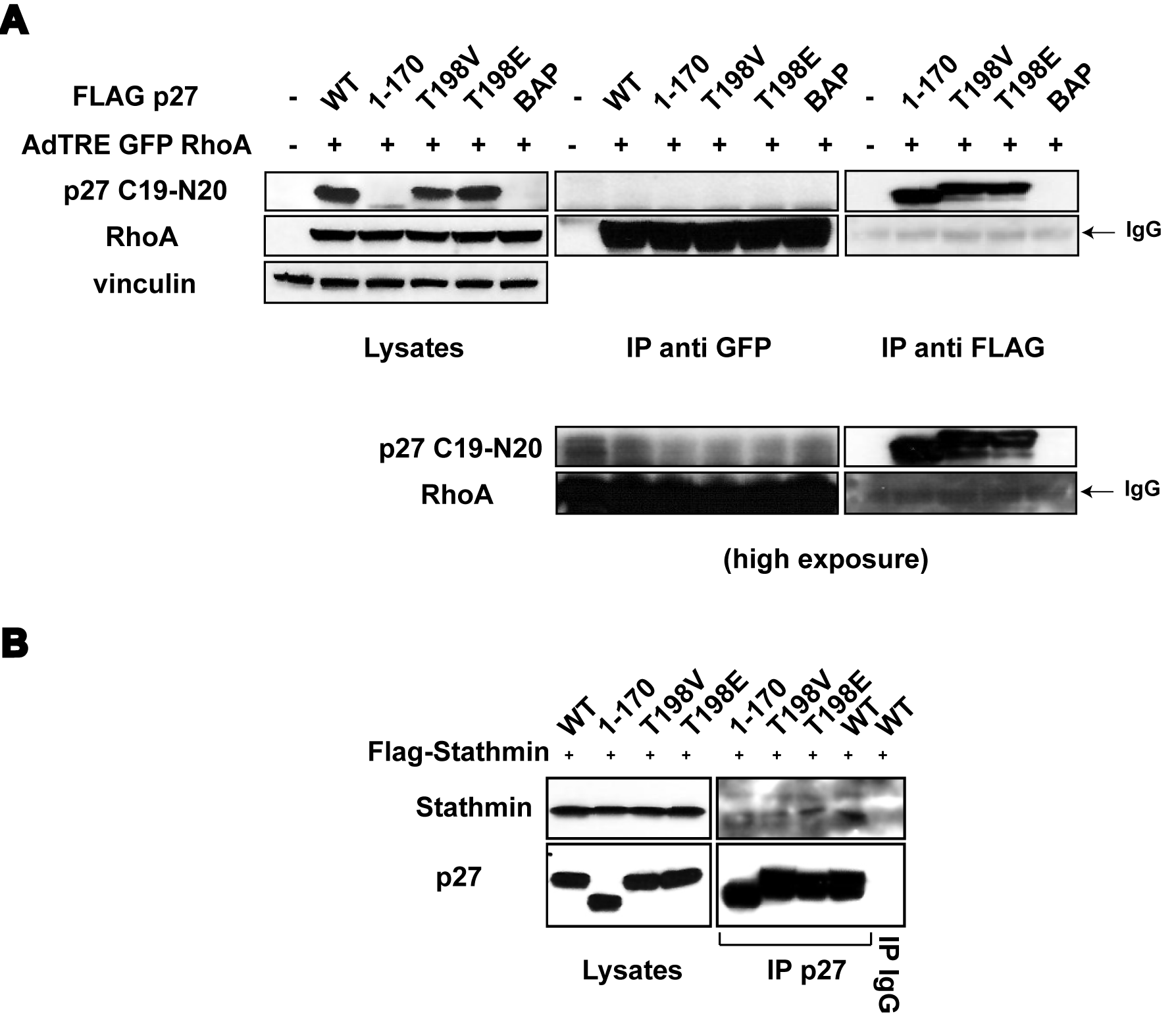

Supplement: Figure S6 — A. Immunoprecipitation (IP) analysis of HT1080 expressing FLAG-p27 and EGFP-RhoA proteins as indicated and adhered on FN for one hour. Total cell lysates and IP proteins were then evaluated by western blot analysis using anti-p27 and anti-Rho antibodies. IPs were performed using both an anti-GFP antibody and the FLAG-M2 affinity gel antibody. No co-precipitation was observed in both the conditions. B. IP analysis of HT1080 expressing p27 and FLAG-stathmin proteins as indicated and adhered on FN for one hour. Total cell lysates and IP proteins were then evaluated by western blot analysis using anti-p27 and anti-Stathmin antibodies. (TIF) [file pone.0017673.s006.tif]
